# Relationship between postpartum depression and plasma vasopressin level at 6–8 weeks postpartum: a cross-sectional study

**DOI:** 10.1038/s41598-022-27223-6

**Published:** 2023-03-02

**Authors:** Masoumeh Kashkouli, Shahideh Jahanian Sadatmahalleh, Saeideh Ziaei, Anoshirvan Kazemnejad, Ashraf Saber, Hamid Darvishnia, Khadijeh Azarbayjani

**Affiliations:** 1 Department of Reproductive Health and Midwifery, Faculty of Medical Sciences, Tarbiat Modares University, Tehran, Iran; 2 Department of Biostatistics, Faculty of Medical Sciences, Tarbiat Modares University, Tehran, Iran; 3 Department of Nursing, Faculty of Medical Sciences, Esfarayen, Iran; 4 Department of Biology, Payame Noor University (PNU), Tehran, Iran

**Keywords:** Health care, Medical research

## Abstract

Postpartum depression (PPD) is the most important postpartum mood disorder due to its significant effect on both the infant and family health. Arginine vasopressin (AVP) has been suggested as a hormonal agent involved in the development of depression. The purpose of this study was to investigate the relationship between the plasma concentrations of AVP and the score of Edinburgh Postnatal Depression Scale (EPDS). This cross-sectional study was conducted in 2016–2017 in Darehshahr Township, Ilam Province, Iran. In the first phase, 303 pregnant women, who were at 38 weeks, met the inclusion criteria, and were not depressed (according to their EPDS scores) were included in the study. In the 6–8 week postpartum follow-up, using the EPDS, 31 depressed individuals were diagnosed and referred to a psychiatrist for confirmation. The maternal venous blood samples of 24 depressed individuals still meeting the inclusion criteria and 66 randomly selected non-depressed subjects were obtained to measure their AVP plasma concentrations with ELISA assay. There was a significant positive relationship between plasma AVP levels and the EPDS score (P = 0.000, r = 0.658). Also the mean plasma concentration of AVP was significantly higher in the depressed group (41.35 ± 13.75 ng/ml) than in the non-depressed group (26.01 ± 7.83 ng/ml) (P < 0.001). In a multiple logistic regression model for various parameters, increased vasopressin levels were associated with increased odds of PPD (OR = 1.15, 95% CI = 1.07–1.24, P = 0.000). Furthermore, multiparity (OR = 5.45, 95% CI = 1.21–24.43, P = 0.027) and non-exclusive breastfeeding (OR = 13.06, 95% CI = 1.36–125, P = 0.026) were associated with increased odds of PPD. Maternal gender preference (having a baby of desired and desired sex) decreased the odds of PPD (OR = 0.13, 95% CI = 0.02–0.79, P = 0.027 and OR = 0.08, 95% CI = 0.01–0.5, P = 0.007). AVP seems to be a contributor to clinical PPD by affecting the hypothalamic–pituitary–adrenal (HPA) axis activity. Furthermore, primiparous women had significantly lower EPDS scores.

## Introduction

Postpartum depression (PPD) is defined as an episode of major depression that is temporally related to the birth of a child^1^. In 2013, the American Psychiatric Association renamed the disorder to "with peripartum onset", stating that the onset of the mood disorder may be during pregnancy or within the first 4 weeks after birth^2^. PPD is different from postpartum blues, which is a mild mood disturbance often occurs within the first 3 to 5 days after delivery^3^. PPD has many negative effects on the mother and newborn; problems that may be brought for the child include developmental disorders, verbal, cognitive and social problems, and the subsequent appearance of behavioral disturbances in the child^4–7^. The causes of PPD are not fully understood^8,9^.

According to The American College of Obstetricians and Gynecologists, perinatal depression affects one out of every seven women^10^. The prevalence of PPD in Iran is reportedly 22% at 6–8 weeks postpartum and 18% at 12–14 weeks postpartum^11^. Many studies support the hypothesis that PPD is related to changes in hypothalamic–pituitary–adrenal axis^12–15^. Vasopressin plays an important role in the stress response and has been identified as an integral part of the hypothalamic–pituitary–adrenal (HPA) axis as a potential factor in stress-related disorders such as anxiety and depression, but the reason why the AVP system is involved in the regulation of the stress response in PPD is yet to be known^16,17^. Many studies have investigated the role of vasopressin in the occurrence of major depression^16–18^. Vasopressin gene is located on chromosome 20^19^ and the effects of vasopressin are mediated by two types of receptors (V1 and V2)^20,21^. Increased activity of the HPA axis in patients with major depression is one of the well-known factors that increase the secretion of corticotropin-releasing hormone (CRH), leading to an increase in secretion of corticotropin and cortisol^22,23^. On the other hand, PPD is a disorder caused by stress conditions^24^, in response to which vasopressin plays a major role in the modulation of HPA axis^13,17^. This hormone, in combination with CRH, can stimulate the release of adrenocorticotropic hormone (ACTH) from the anterior lobe of pituitary, and eventually, cause the release of cortisol/corticosterone from the adrenal gland^25,26^. Animal studies have shown that in depressed cases, vasopressin, instead of CRH, has a major stimulatory role on ACTH secretion^26^. There is evidence indicating that such a role for vasopressin is also conceivable in humans. For example, in depressed individuals, the number of AVP-immunoreactive neurons increases in the paraventricular nucleus (PVN), which can potentiate the effects of CRH^27^.

Given the possibility of a relationship between AVP and depression^16–18^, it seems necessary to investigate its relationship with PPD. To our knowledge, no original research has directly examined the relationship yet. The present study aimed to seek for (1) a possible relationship between plasma AVP levels and the Edinburgh Postnatal Depression Scale (EPDS) score at 6–8 weeks postpartum, and (2) a possible relationship between PPD and factors like parity, BMI, maternal gender preference, type of delivery, gender of the baby, maternal age, history of abortion, women’s education level, husband’s education level, and breastfeeding status at 6–8 weeks postpartum.

The hypotheses of the study were as follows: (1) There is a positive relationship between plasma AVP levels and EPDS score at 6–8 weeks postpartum, so depressed women have higher plasma levels than non-depressed women at 6–8 weeks postpartum. (2) There is a significant association between PPD and factors like parity, BMI, maternal gender preference, type of delivery, gender of the baby, maternal age, history of abortion, women’s education level, husband’s education level, and breastfeeding status at 6–8 weeks postpartum.

## Material and methods

The present cross-sectional study was conducted within 2016–2017.

### Participants

The required sample size to study on pregnant women at 38 weeks of gestation was estimated to be 303 individuals (CI = 95%, P-value = 5%). For assessing the relationship between plasma vasopressin level and EPDS score at 6–8 weeks postpartum, the sample size was calculated to be 95 persons. All the 303 subjects were selected from among those who were referred to Urban and Rural Health Care Centers for prenatal care and were not depressed according to their EPDS score (< 13). They were first briefed about the study objectives and confidentiality of maternal and neonatal information, and then their informed consent was obtained.

The study inclusion criteria were singleton pregnancy, no systemic diseases such as lupus and diabetes mellitus, no pregnancy complications like diabetes, pre-eclampsia, etc., no previous history of psychological problems, Iranian nationality, no use of antidepressants, hormonal contraceptive pills, or sleeping pills within 2 weeks prior to venous blood sampling, good marital relationship with the spouse, no expressed significant economic problems, and no family history of depression or other mental illnesses. The study exclusion criteria were experiencing stressful conditions or using alcohol within 12 h before sampling, insufficient sleep and heavy physical activity the night before sampling, abnormal blood pressure during sampling or at postpartum period, instrumental vaginal delivery, congenital malformations of the newborn, and complications during childbirth (vaginal delivery or cesarean section) leading to treatments such as blood transfusion, resuscitation, hospitalization in the ICU or CCU, or transfer to the operating room. The mothers were controlled according to the routine prenatal care program until delivery. All participants (n = 303) were once again assessed with the Edinburgh Questionnaire during 6 to 8 weeks after delivery, and if they received a score of 13 or higher, they were referred to a psychiatrist to confirm their depression. Thirty-one of them scored 13 or more; of which, PPD of 29 subjects was confirmed by the psychiatrist. Sixteen non-depressed and five depressed subjects did not meet one of the inclusion criteria or were excluded from the study. Finally, the number of subjects in the depressed and non-depressed groups were 24 and 66, respectively.

Methods of data collection included observation, examination (weight, height, BMI, and other criteria in prenatal care forms such as blood pressure, fetal heart rate, fundal height, and warning signs during pregnancy), and patient interview. Gestational age was calculated from the first day of the last menstrual period (LMP), or the first trimester ultrasound (if uncertain about LMP). Weight, blood pressure, and heart rate of the fetus were measured by the same person using a digital scale, digital barometer, and fetal heart detector (Sonicaid), respectively.

### Questionnaires

The patient interview was conducted using a questionnaire including three sections of personal information, obstetrics and medical history, and laboratory results. All the data were finally recorded in a checklist. Another questionnaire used in this study was the 10-item EPDS, designed by Cox et al.^28^, to evaluate maternal depression at 38 weeks of gestation and 6–8 weeks postpartum. Each question is given a score of 0 to 3. We used the Persian (Iranian language) version of the EPDS. The acceptability, reliability, and validity of the EPDS have been verified by Montazeri et al.^11^. Cut-off point was defined as 13 or greater.

### Determination of vasopressin level

Venous blood samples were taking from all the 24 depressed and 66 randomly selected non-depressed individuals 6 to 8 weeks after delivery at 9–9:30 h. Blood samples were taken after 15-min rest. The samples were added to the EDTA-containing chilled plastic tubes. Then they were immediately kept at 4 °C within 30 min, and plasma separation was carried out. The samples were centrifuged at 3000 rpm for 10 min at 4 °C. The produced plasma was frozen at a temperature of − 80 °C until analysis. After transferring to the Endocrinology and Metabolism Laboratories of Shahid Beheshti University of Medical Sciences, Tehran/Iran, the plasma vasopressin level was measured by the ELISA method using Human Anti-Diuretic Hormone (ADH) ELISA kit (ZellBio GmbH, Ulm Germany) with a sensitivity of 0.5 ng/ml. In our study, plasma osmolality was not measured.

The relationship between plasma vasopressin levels and the EPDS score was assessed. Then the participating mothers were divided into depressed and non-depressed groups according to the EPDS score and confirmation by psychiatrist. Next, the mean plasma AVP level was compared between the two groups. Finally, a binary logistic regression analysis was carried out to further understand the association of the independent variables, including AVP and clinical-anamnestic factors with PPD. Odds ratio with 95% confidence interval was used.

### Statistic

The normal distribution of variables in each group was assessed using the Kolmogorov–Smirnov test. A Chi-squared test was used for qualitative variables when comparing the groups. Mann–Whitney test and Independent-sample T-test were applied for non-normally and normally distributed variables, respectively. Binary logistic regression was employed to assess the possible association of PPD with the variables like plasma vasopressin levels, parity, BMI, maternal gender preference, type of delivery, parity, maternal age, history of abortion, women’s education level, husband’s education level and breastfeeding status. For all statistical tests, the level of significance was considered as P < 0.05. Data analysis was done using the SPSS software (ver. 21).

### Ethics approval and consent to participate

The study protocol was approved by the Institutional Review Board and the Ethics Committee of Tarbiat Modares University of Medical Sciences (IR.TMU.REC.1394.182). All procedures were in accordance with the ethical standards of the Regional Research Committee and with the Declaration of Helsinki 1964 and its later amendments. After explaining the research purposes, a written consent and a verbal assent were collected from all participants. They were also informed that their participation was voluntary, confidential and anonymous, and that they had the right to withdraw from the research at any time.

## Results

Demographic and background data for the two groups are presented in Table 1. Overall, 90 participants were included in the present study at 6–8 weeks postpartum. The results showed that 24 women had depression and 66 women had no depression. There was not a significant difference in the mean of maternal age and BMI between the depressed and non-depressed groups (P > 0.05).

**Table 1:** Demographic and clinical characteristics of the subjects (n = 90).

Characteristics		Individual status		P-value
		Depressed (n = 24)	Non-depressed (n = 66)	
		(N) %	(N)%	
Women’s education	Less than high school diploma	4 (16.7)	8 (12.1)	0.37
	High school diploma	12 (50)	25 (37..9)	
	University	8 (33.3)	33 (50)	
Husband’s education	Less than high school diploma	4 (16.7)	9 (13.6)	0.79
	High school diploma	11 (45.5)	27 (40.9)	
	University	9 (37.5)	30 (45.5)	
History of abortion	Yes	1 (4.2)	8 (12.1)	0.43
	No	23 (95.8)	58 (87.9)	
Maternal gender preference	Desired sex	4(33.3)	20(41.7)	0.63
	Non-desired sex	8 (66.7)	28 (58.3)	
Baby gender	Male	11 (45.8)	34 (51.5)	0.63
	Female	13 (54.2)	32 (48.5)	
Mode of delivery	NVD	9 (25)	27 (75)	0.77
	CS	15 (27.8)	39 (72.2)	
Parity	Nulliparous	9 (37.5)	39 (59.1)	0.06
	Multiparous	15 (62.5)	27 (40.9)	
Breastfeeding status at 6–8 week postpartum	No exclusive breastfeeding	6 (25)	2 (3)	0.004
	Exclusive breastfeeding	18 (75)	64 (97)	
Living area	City	17 (70.8)	51 (77.3)	0.53
	Village	7 (29.2)	15 (22.7)	

### Prevalence of PPD

A total of 303 women at 38 weeks of pregnancy from the Urban and Rural Health Care Centers met the inclusion criteria. The mean score of EPDS scale was higher in the depressed women (16.46 ± 3.10) as compared with the non-depressed women (6.12 ± 13.32) (P < 0.001). Finally, after confirmation of PPD by the psychiatrist, the prevalence of PPD was determined as 9.57%.

### Association of the plasma concentrations of AVP and clinical- anamnestic factors with PPD

The Pearson’s correlation test showed a positive significant relationship between the plasma concentrations of AVP and EPDS score in the depressed group (P < 0.001, r = 0.65). According to the Independent-sample T test results, the mean plasma concentrations of AVP was significantly higher in the depressed group (41.35 ± 13.75 ng/ml) than in the non-depressed group (26.01 ± 7.83 ng/ml) (t (28.6) = 5.16, P < 0.001). We performed binary logistic regression to investigate the association of plasma vasopressin levels and clinical-anamnestic factors, (including parity, BMI, maternal gender preference, type of delivery, gender of the baby, parity, maternal age, history of abortion, women’s education level, husband’s education level and breastfeeding status) with PPD. After analysis, plasma vasopressin levels, parity, maternal gender preference and breastfeeding status showed statically significant relationship with PPD. In the final prediction model, the increasing values of plasma vasopressin corresponded with increasing of the odds of PPD (OR = 1.15, 95% CI = 1.07–1.24, P < 0.001).

The association between the clinical-anamnestic factors, including mode of delivery (P = 0.77), gender of the baby (P = 0.63), living area (p = 0.53), women’s education level (P = 0.37), husband’s education level (P = 0.79), maternal gender preference (P = 0.63), parity (P = 0.06), gender of the baby (P = 0.63), history of abortion (P = 0.43), breastfeeding status (P = 0.004) and PPD was quantified by Chi-squared test. Among the above variables, only breastfeeding status was related to PPD (P = 0.004) (Table 1).

Using binary logistic regression analysis, the non-exclusively breastfeeding women were found to experience PPD 13 times more than the exclusively breastfeeding (OR = 13.06, 95% CI = 1.36–125, P = 0.026). The exclusively breastfeeding women were divided into depressed and non-depressed groups. We ran a Mann–Whitney’s U test because of the abnormal distribution of AVP levels in the non-depressed group to evaluate the difference in the concentrations of AVP in the exclusively breastfeeding depressed and non-depressed women. The mean plasma AVP concentrations in the depressed and non-depressed groups were 66.73 and 37.78 ng/ml, respectively, showing a significant difference (U = 282, Z = − 4.64, P < 0.001).

An independent T-test was conducted to compare the mean of maternal age between the two groups. There was not a significant difference in the mean of maternal age between the depressed and non-depressed groups.

Multiparous (OR = 5.45, 95% CI = 1.21–24.43, P = 0.027) were more likely to be depressed. Women with no gender preference were more likely to be depressed than ones who had a baby of the non-desired (OR = 0.13, 95% CI = 0.02–0.79, P = 0.027) and desired (OR = 0.08, P = 0.007, 95% CI = 0.01–0.5) sex (Table 2).

**Table 2:** Logistic regression analysis of factors affecting postpartum depression.

Predictors		Adjusted OR	CI95%	P-value*
Vasopressin		1.15	1.07–1.24	< 0.001
Parity	Primiparous	1		
	Multiparous	5.45	1.21–24.43	0.027
Breastfeeding status	Exclusive	1		
	Non-exclusive	13.06	1.36–125	0.026
Maternal gender preference	No preference	1		
	Desired sex of the baby	0.08	0.01–0.0.5	0.007
	Non-desired sex of the baby	0.13	0.02–0.79	0.027

R2 = 0.41.

*P values are adjusted for education level, history of abortion, baby gender, mode of delivery, and living area.

## Discussion

The main aim of this study was to investigate the association between PPD and the plasma concentrations of AVP. Based on our findings, the prevalence of PPD (as confirmed by the psychiatrist) was 9.57% at 6–8 weeks postpartum.

In the present study, there was a significant positive correlation between the mean plasma concentration of AVP and EPDS score; the mean plasma concentration of AVP was significantly higher in the depressed group than in the non-depressed group. Worldwide epidemiological studies show that depression occurs in women twice as much as in men and peaks during the first year postpartum. A few previous studies on the relationship between AVP and depression in non-pregnant populations are as follows. In a 2006 review article, Keck concluded that a dysfunction in AVP and corticotropin-releasing factor (CRF) systems is involved in the pathogenesis and etiology of depression and anxiety^29^. Increased plasma levels of AVP were suggested in depressed patients^30^. In the study of Van Londen et al., the mean plasma concentration of AVP was higher in the depressed patients than in the control group^18^. In chronic stress, vasopressin level is steadily elevated in CRH neurons so that even a slight irritation may lead to a severe AVP/CRH secretion. This can contribute to the development of dysphoric symptoms and may even be a factor responsible for the onset and permanent characteristics of major depression^31^. The number of CRH neurons, co-express vasopressin, increases in depressed human PVN^32^. Elevated AVP mRNA level in the supraoptic nucleus (SON) of depressed individuals^33^ also leads to an increase in plasma vasopressin level^26^, which is related to suicide risk^34^ and altered attention and arousal in memory processes in these patients^35^. In contrast, Wang et al. found no significant difference in vasopressin mRNA expression in the SON or PVN of depressed individuals comparing to the control group, probably due to the mere presence of melancholic depressed individuals in this study^36^. Brunner et al. could not find any association between vasopressin concentration in plasma and cerebrospinal fluid (CSF) among the depressed and non-depressed individuals. The two were also not different in those who attempted suicide compared to other people^37^. This finding is not in line with the results of the present study. It might be due to low sample size and diagnostic heterogeneity in the patient and control groups in the above study, which did not allow them to investigate the pathogenetic role of vasopressin.

Griebel et al. suggested that although CRF has been recognized as a key regulator of the stress system, there is evidence that the vasopressinergic system may play an equal role in regulating the stress responses; so vasopressin V (1b) receptor antagonists may be potential treatment for depression. This finding confirms the efficacy of using SSR149415, which was the first selective, orally active vasopressin V (1b) receptor antagonist in the treatment of depression and anxiety disorders caused by traumatic events^38^. Vasopressin V (1b) receptor is required for the normal response of the HPA axis during chronic stress^39^. Muller et al.’s study on mice showed a selective compensatory activation of the hypothalamic vasopressinergic system for maintaining basal ACTH secretion and HPA system activity in heterozygous and homozygous Crhr1−/− mutants in basal and stress conditions. Deficiency of CRH receptor 1 (CRHR1) severely impairs the stress response of the HPA system and reduces stress-related behavior in mice^40^.

The research findings revealed a significant positive relationship between the plasma concentrations of AVP and the EPDS score at 6 to 8 weeks postpartum. The mean plasma concentrations of AVP were significantly higher in the depressed group (41.35 ± 13.75 ng/ml) than in the non-depressed group (26.01 ± 7.83 ng/ml) (t (28.6) = 5.16, P < 0.001). In this study, all subjects were controlled for the involvement of other factors affecting plasma vasopressin concentration. The obtained results support the hypothesis of a relationship between AVP and PPD. Since PPD is classified as a sub-type of major depressive disorder, our findings also support the possible role of vasopressin in the development of major depression.

According to the logistic regression results, primiparous status was a significant negative predictor of depression. Some studies show that primiparous women are more anxious during pregnancy comparing to the postpartum period. Our study further confirms that since most of primiparous women are happy with the birth of a newborn and addition of a new member to the family, their EPDS score lower comparing to other women. In a study by Righetti-Veltema et al. in Geneva on the risk factors and predictive signs of PPD at 3 months after delivery, the multiparas reported more difficult pregnancies and higher anxiety and were less involved in the perinatal preparation program in comparison to the control group^41^. Figueiredo and Conde reported that parity has a significant impact on postpartum anxiety and PPD. According to their study, second-time parents showed more anxiety and depression symptoms than first-time parents in the second and third trimesters and also 3 months after childbirth, but not at birth^42^. These results are in line with our results. In contrast, O'Hara et al. reported that pregnant and primiparous women experienced more depressive symptoms, marital distress, and psychological problems than non-pregnant women^43^. In Kaij et al.’s study, symptoms of depression decreased (using the EDPS scale) with the increasing number of children^44^. Gotlib et al. showed that multiparity was a risk factor for depression during pregnancy, but not in the postpartum period^45^. Wenzel et al. claimed that there was typically no relationship between parity and depression during pregnancy and postpartum^46^, which is consistent with the findings of Lashkaripour et al.^47^. None of the latter four studies could make a clear and well-established conclusion regarding the relationship between primiparous status and PPD. These conflicting results can be due to different tools used for PPD assessment or different times of evaluation. There are also unclear results about the role of other factors affecting the relationship between parity and PPD such as parents’ education level, socioeconomic factors, and maternal age. We excluded people who were economically disadvantaged to control this variable.

In the present study, 60 women (66.6% of all participating women) had gender preference; of which, 40% had a baby of the non-desired sex and only 20% of them developed PPD. In general, only 16.6% of all PPD women had a baby of the non-desired sex. The majority of the PPD women had not gender preference. Depression in the two groups of women whether their baby had the same gender as they desired before birth (or not) was less than in the group of women who did not have preference about the sex of the baby. Preference of boy baby is still common in the countries like India, China and some other parts of the world, which is deeply rooted in their cultural issues. In these societies, the girl is considered merely an economic consumer of the family as most girls get married, are paid dowry, and then leave the family^48,49^. In one study, having a baby of the non-desired sex increased the risk of developing PPD^50^. Although the results of these studies contradict our findings (Table 2), similar to ours, Dhillon and MacArthur identified that maternal gender preference in Asian women living in the UK had no association with PPD^51^.

Within the total research sample at 6–8 weeks postpartum, 82 women breastfed exclusively (91.1%) and 8 women did not initiate or ceased exclusive breastfeeding. In our study, exclusive breastfeeding included Labbock and Krasovec’s levels 1 and 2^52^. A number of studies have reported no relationship between breastfeeding and PPD^53^ while some others have shown that breastfeeding may protect against PPD^52,53^; the latter is consistent with the present study results, suggesting that exclusive breastfeeding may help to reduce mother’s PPD. In our study, women who exclusively breastfed their neonates at 6–8 weeks postpartum were less likely to be depressed.

## Study limitations

This study provides useful data on the relationship between plasma AVP and PPD. However, we had some limitations in conducting the study. For example, it would be better to evaluate plasma concentrations of AVP by Radioimmunoassay (RIA). However, we could not do it because of lack of access to Human Vasopressin Radioimmunoassay (RIA) kits in Iran due to sanctions.

## Conclusion

To the best of our knowledge this is the first paper on the relationship between maternal plasma AVP levels and PPD. The results revealed that the mean plasma AVP level was higher in the depressed group comparing to the non-depressed group. Also the mean plasma concentration of AVP was higher in the exclusively breastfed depressed women comparing to their non-depressed counterparts. Additionally, being a primiparous, exclusive breast-feeding and maternal gender preference were negatively related to PPD. Vasopressin appears to be related to PPD by contributing to the dysregulation of the HPA axis. It is recommended that future studies be conducted with larger sample sizes and longer follow-up periods.

## Data Availability

The data sets used and analyzed for the current study are available upon reasonable request.
